# Oxygenation strategies prior to and during prehospital emergency anaesthesia in UK HEMS practice (PREOXY survey)

**DOI:** 10.1186/s13049-020-00794-x

**Published:** 2020-10-12

**Authors:** Adam J. Boulton, Amar Mashru, Richard Lyon

**Affiliations:** 1 Warwick Medical School, University of Warwick, Coventry, UK; 2 Academic Department of Anaesthesia, Critical Care, Pain and Resuscitation, Heartlands Hospital, University Hospitals Birmingham NHS Foundation Trust, Birmingham, B9 5SS UK; 3 Air Ambulance Kent Surrey Sussex, Rochester Airport, Maidstone Road, Chatham, Rochester UK; 4 University of Surrey, Stag Hill, Guildford, UK

**Keywords:** Pre-hospital emergency anaesthesia, Rapid sequence induction, Pre-oxygenation, Apnoeic oxygenation, Airway management, Emergency medical services

## Abstract

**Background:**

Maintaining effective oxygenation throughout the process of Pre-Hospital Emergency Anaesthesia (PHEA) is critical. There are multiple strategies available to clinicians to oxygenate patients both prior to and during PHEA. The optimal pre-oxygenation technique remains unclear, and it is unknown what techniques are being used by United Kingdom Helicopter Emergency Medical Services (HEMS). This study aimed to determine the current pre- and peri-PHEA oxygenation strategies used by UK HEMS services.

**Methods:**

An electronic questionnaire survey was delivered to all UK HEMS services between 05 July and 26 December 2019. Questions investigated service standard operating procedures (SOPs) and individual clinician practice regarding oxygenation strategies prior to airway instrumentation (pre-oxygenation) and oxygenation strategies during airway instrumentation (apnoeic oxygenation). Service SOPs were obtained to corroborate questionnaire replies.

**Results:**

Replies were received from all UK HEMS services (*n* = 21) and 40 individual clinicians. All services specified oxygenation strategies within their PHEA/RSI SOP and most referred to pre-oxygenation as mandatory (81%), whilst apnoeic oxygenation was mandatory in eight (38%) SOPs. The most commonly identified pre-oxygenation strategies were bag-valve-mask without PEEP (95%), non-rebreathable face mask (81%), and nasal cannula at high flow (81%). Seven (33%) services used Mapleson C circuits, whilst there were eight services (38%) that did not carry bag-valve-masks with PEEP valve nor Mapleson C circuits. All clinicians frequently used pre-oxygenation, however there was variability in clinician use of apnoeic oxygenation by nasal cannula. Nearly all clinicians (95%) reported manually ventilating patients during the apnoeic phase, with over half (58%) stating this was their routine practice. Differences in clinician pre-hospital and in-hospital practice related to availability of humidified high flow nasal oxygenation and Mapleson C circuits.

**Conclusions:**

Pre-oxygenation is universal amongst UK HEMS services and is most frequently delivered by bag-valve-mask without PEEP or non-rebreathable face masks, whereas apnoeic oxygenation by nasal cannula is highly variable. Multiple services carry Mapleson C circuits, however many services are unable to deliver PEEP due to the equipment they carry. Clinicians are regularly manually ventilating patients during the apnoeic phase of PHEA. The identified variability in clinical practice may indicate uncertainty and further research is warranted to assess the impact of different strategies on clinical outcomes.

## Background

Endotracheal intubation allows definitive protection of the airway and may optimise oxygenation and ventilation in critically unwell patients [1, 2]. Advanced airway interventions, including Pre-Hospital Emergency Anaesthesia (PHEA), are being increasingly performed in the pre-hospital environment [3–6] with high procedural success [7, 8]. However, severe complications are associated with PHEA including hypoxaemia and cardiovascular instability [9–12]. One technique to minimise the risk of hypoxaemia is to provide the patient with a period of pre-oxygenation [13]. Pre-oxygenation is recommended by multiple anaesthetic guidelines in a variety of clinical settings, including pre-hospital care [14–18]. A period of successful pre-oxygenation significantly delays the onset of desaturation during apnoea by wash-out of nitrogen from the lungs, increased oxygen content in the lungs, and an increased oxygen tension in the blood and tissues [13, 19]. Patients undergoing PHEA in the pre-hospital setting are clinically unstable and regularly have airway, respiratory and haemodynamic compromise and consequently pre-oxygenation may be ineffective [20]. Given the deranged physiology, efforts to optimise pre-oxygenation are of heightened clinical significance [10, 20]. However, reliably performing adequate pre-oxygenation in critically unwell patients can be problematic due to poorly co-operative patients, concerns over gastric insufflation in an unfasted patient and equipment/logistical concerns [14, 21, 22]. There are multiple pre-oxygenation techniques available including oxygen delivery by nasal cannula, facemask, non-invasive ventilation, bag valve mask (BVM), Mapleson C circuit, or high-flow nasal cannula [11, 19, 21, 23]. The optimal technique in the pre-hospital arena is unclear and anecdotally clinicians are using a variety of techniques [14]. As such, it is uncertain which pre-oxygenation techniques are currently being employed by UK Helicopter Emergency Medical Services (HEMS) and if there is a variability between services. Although defined inconsistently in the present literature, apnoeic oxygenation may be used as an adjunct to pre-oxygenation by a range of methods [24–27]. Similarly, its application in contemporary UK HEMS practice is unknown [3]. Identification of the oxygenation strategies being used in UK HEMS practice will allow better understanding of current practice and inform development of future observational or interventional studies. Therefore, the aim of this study was to identify the current oxygenation strategies used prior to and during PHEA by UK HEMS services and individual clinicians.

## Methods

The Checklist for Reporting Results of Internet E-Surveys (CHERRIES) has been followed in the reporting of this electronic survey [28]. A cross-sectional questionnaire survey hosted by Google Forms was distributed by email to all UK HEMS services between 05 July and 26 December 2019. Questionnaires were developed to investigate service standard operating procedures (SOP) and individual clinician practice. The questionnaires were developed with pre-hospital care experts and were informed by the oxygenation strategies described in the updated Utstein-style pre-hospital advanced airway template [29]. So that responses regarding oxygenation strategies were reliable, care was taken in clearly defining oxygenation strategies through PHEA. A practical definition to limit subjectivity was used, with the point of airway instrumentation used as the point of division. Questions investigated oxygenation strategies prior to airway instrumentation (pre-oxygenation) and oxygenation strategies during airway instrumentation (apnoeic oxygenation). Piloting of the questionnaire with pre-hospital experts and iterative refinement minimised question ambiguity. The questionnaires are found in Supplementary Material 1. The first questionnaire aimed to assess the local service SOP for PHEA and oxygenation strategy described. A reply regarding the service SOP along with a copy of the service SOP to corroborate findings was sought from every UK HEMS service (*n* = 21). The second questionnaire aimed to investigate individual clinician practice. Replies were sought from two individual clinicians at each service, ideally a HEMS consultant and a HEMS junior doctor, but in services with no HEMS junior doctor, responses were sought from one senior consultant and one junior consultant. Questions were asked in the same order to all participants and required fields ensured only complete questionnaires could be submitted.

Data were summarised using standard descriptive statistics and graphical plots. Data are reported anonymously, and individual HEMS services are not be identifiable in published results. Responses were compiled and analysed using Microsoft Excel.

This survey met UK NIHR criteria as a service evaluation and therefore formal Health Research Authority approval was not required [30]. No patient identifiable data was collected.

## Results

### HEMS service standard operating procedures

**Fig. 1:**
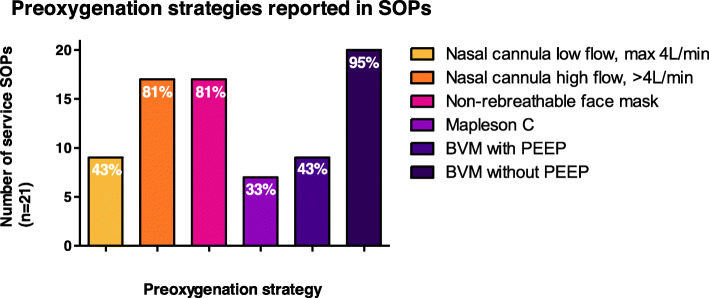
Preoxygenation strategies reported in SOPs. BVM = Bag valve mask, PEEP = Positive End Expiratory Pressure, SOP = Standard Operating Procedure

**Fig. 2:**
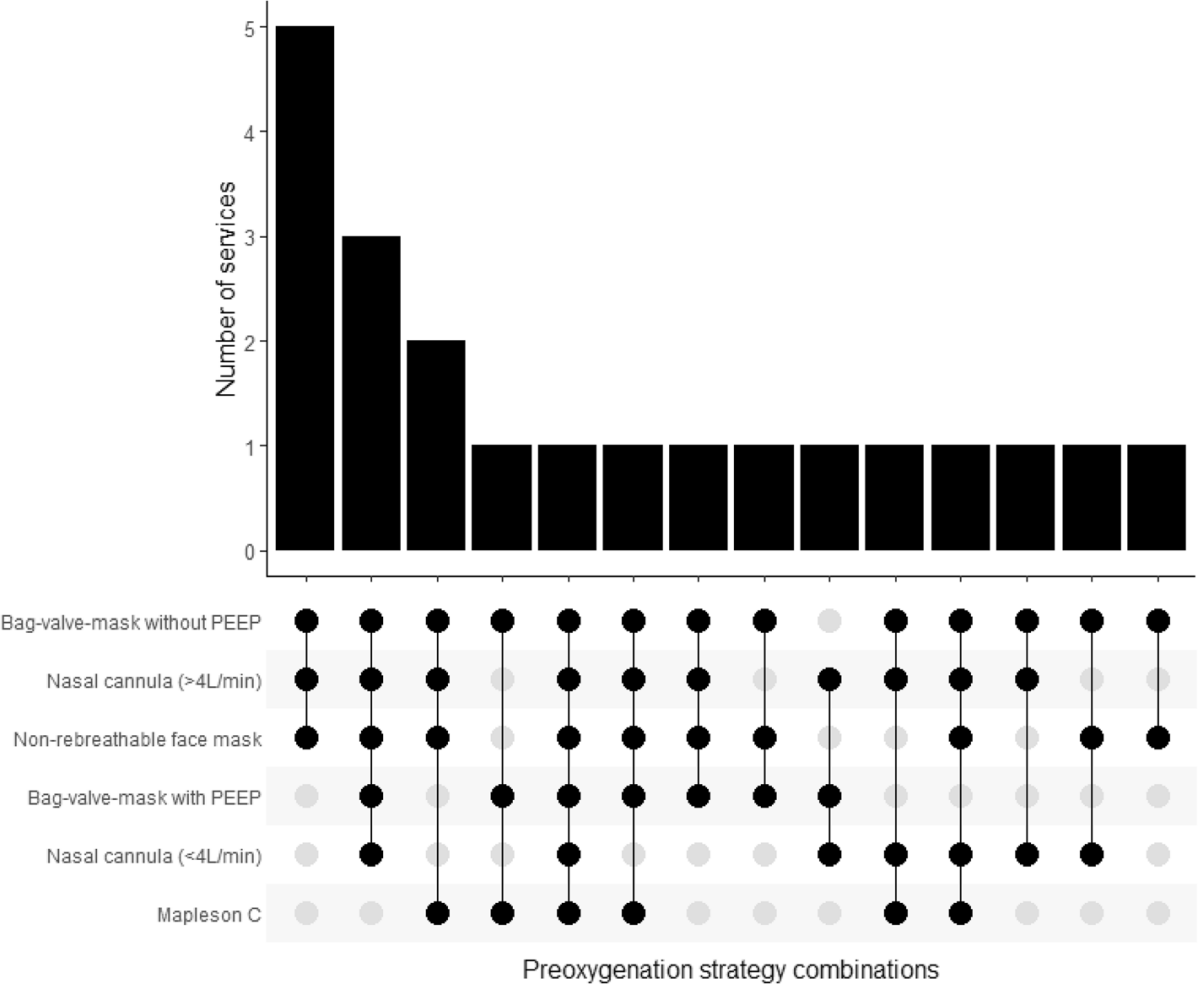
Combinations of preoxygenation strategies reported in SOPs

Reponses were received from every UK HEMS service (*n* = 21). A copy of the local service SOP was received from 13 services and used to corroborate questionnaire responses. A doctor-paramedic model was used by the majority of services when delivering PHEA (*n* = 20, 95%) with one service employing a paramedic-paramedic model. The approximate number of episodes of PHEA was variable across services with 12 (57%) performing > 100 per year, 6 (29%) performing 50–100 per year, and 3 (14%) < 50 per year. All services had a PHEA/RSI SOP and pre-oxygenation was referred to in this SOP by all services. No service had a separate SOP for oxygenation during PHEA. Most service SOPs referred to pre-oxygenation as mandatory (*n* = 17, 81%) and the remainder referred to pre-oxygenation as advised (*n* = 4, 19%). No SOPs identified specific patient groups in which pre-oxygenation should be used. There was a range of pre-oxygenation strategies referred to in service SOPs. The most common strategies were BVM without PEEP (*n* = 20, 95%), non-breathable face mask (*n* = 17, 81%), and nasal cannula at high flow (> 4 L/min) (n = 17, 81%) (Fig. 1). Specific combinations of SOP pre-oxygenation strategies varied across services (Fig. 2). The most common combination was that of BVM without PEEP, non-rebreathable face mask, and nasal cannula at high flow (> 4 L/min) (*n* = 5, 24%). There were eight services (38%) that did not specify BVM with PEEP valve nor Mapleson C circuit within their SOP, therefore precluding delivery of PEEP. Ten service SOPs (48%) recommended use of a BVM with PEEP valve or a Mapleson C circuit, and three services (14%) identified both devices. Seven services (33%) carried Mapleson C circuits, all of whom also carried BVMs. No service SOP referred to non-invasive ventilation as a possible pre-oxygenation strategy.

Apnoeic oxygenation was discussed in all but one PHEA SOP (*n* = 20, 95%). Apnoeic oxygenation was referred to as mandatory by eight (38%), advised by seven (33%), and to be considered by 5 (24%) SOPs. Two service SOPs (10%) identified patient groups where apnoeic oxygenation should be considered, and these were obese patients and predicted airway difficulties. All service SOPs noting apnoeic oxygenation referred to nasal cannula, with 16 (76%) stating it should be at high flow (> 4 L/min) and the reminder not specifying a recommended flow.

### Individual clinician practice

Doctors from every HEMS service were invited to complete questionnaires investigating their individual practice. Forty participants completed the individual questionnaire covering all HEMS services. Responses from a senior consultant and a junior doctor were received from 15 services. Four services did not have junior doctors, and hence replies were sought from one senior consultant and one junior consultant. Two services did not have a junior consultant and therefore replies were gained from one senior consultant. Questionnaire responses were received from 25 HEMS consultants and 15 HEMS junior doctors. The years of experience as a doctor ranged from 8 to 22 years (median 13, IQR 4.3) and years of experience in HEMS ranged from 1 to 20 years (median 4.5, IQR 8).

Thirty-six participants (90%) stated they always used pre-oxygenation and four (10%) stated they used pre-oxygenation very often (75–100% of cases). The pre-oxygenation strategies individuals reported they frequently used were similar to those identified in service SOPs (Table 1). No clinician reported use of non-invasive ventilation, in keeping with service SOPs.

**Table 1:** Pre-oxygenation strategies

Pre-oxygenation strategy frequently used by individuals	N	Percentage of respondents (*n* = 40)
Bag Valve Mask without PEEP	22	55.0%
Bag Valve Mask with PEEP	7	17.5%
Mapleson C	13	32.5%
Non-rebreathable face mask	17	42.5%
Nasal cannula, high flow (> 4 L)	17	42.5%
Nasal cannula, low flow (max 4 L)	7	17.5%
Nasal cannula, but flow not specified	1	2.5%

Three participants (8%) stated they were not able to deliver their preferred pre-oxygenation strategy in their current service. These all related to inability to deliver PEEP and participants stated the desire to have a BVM with PEEP valve or Mapleson C available. The service SOPs these clinicians worked within showed that the service did not carry either a BVM with a PEEP valve or Mapleson C and hence PEEP could not be delivered.

**Fig. 3:**
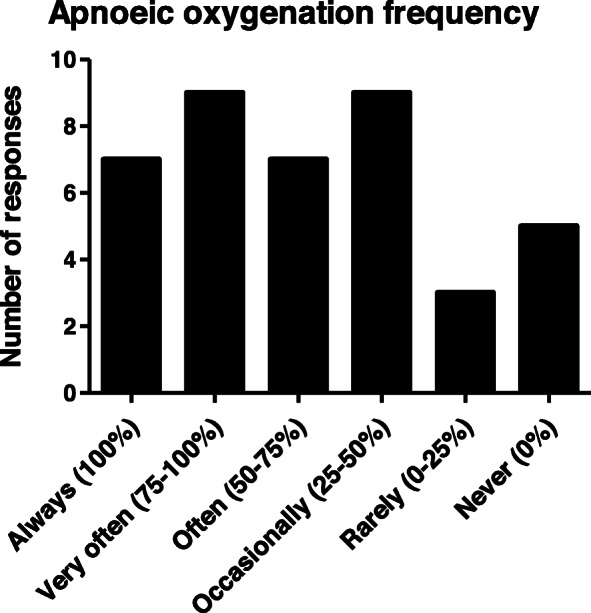
Individual clinician use of apnoeic oxygenation

In contrast to pre-oxygenation, there was a large variation in how frequently individuals use apnoeic oxygenation during PHEA. There was an almost equal distribution across frequency of clinician use of apnoeic oxygenation in their last five deliveries of PHEA (Fig. 3).

Of the 35 participants (88%) who reported apnoeic oxygenation use, the majority used nasal cannula at high flow (≥4 L/min) (*n* = 32, 91%) and the remainder at low flow (max 4 L/min) (n = 3, 9%). Three participants stated that they were unable to deliver their preferred apnoeic oxygenation strategy, which was humidified high flow nasal oxygenation such as THRIVE or OptiFlow™.

We asked clinicians to describe how their oxygenation strategy changed after delivery of anaesthetic drugs but before airway instrumentation. Over half of participants (*n* = 23, 58%) stated they routinely ventilate patients during the apnoeic phase. A further 15 (38%) stated they would occasionally ventilate during the apnoeic phase dependent on the clinical situation, such as when the patient showed prior signs of ventilatory failure, was deemed at high risk of desaturation, or if the oxygen saturations dropped. Therefore, 95% of participants reported ventilating patients during the apnoeic phase when performing PHEA. Two participants (5%) reported no change in oxygenation strategy after delivery of anaesthetic drugs but before airway instrumentation. Twelve participants (30%) stated they increase oxygen flow via nasal cannula from low flow to high flow (> 4 L/min).

When comparing their overall oxygenation strategies between PHEA and in-hospital practice, 14 participants (35%) reported there was no difference. In the remainder, differences between PHEA and in-hospital technique were use of humidified high flow nasal oxygenation such as THRIVE or OptiFlow (*n* = 14, 35%), and use of a Mapleson C circuit (n = 14, 35%).

## Discussion

Our study set out to provide a snapshot of current PHEA practices across UK HEMS services, with a particular focus on pre-oxygenation and apnoeic oxygenation strategies. When evaluating the merits, drawbacks and lessons learned from this study, it is worth noting initially that the survey was answered by every HEMS in the UK. This provides us not only with a valuable insight into the makeup of our HEMS around the country, their utilisation of SOPs and their rates of PHEA, but as a springboard for sharing, comparing and understanding practice such that it can be enhanced for the benefit of the specialty and the patients it serves.

More than 100 PHEA episodes were undertaken each year by the majority of services, with PHEA being detailed by every service in a dedicated SOP. Pre-oxygenation strategies were widely implemented during PHEA and were mentioned in every SOP. However, it is worth noting that 20% of services did not mandate its use. Individuals appeared more universally keen to utilise pre-oxygenation, but it was still not implemented 100% of the time in those surveyed. This study is not intended to debate the pros and cons of such practice, instead we see this as a valuable example of the purpose of work such as ours in challenging or supporting assumptions that may exist regarding PHEA. We noted a spread of modalities for delivering preoxygenation are currently utilised, with very few respondents feeling they were unable to deliver their preferred pre-oxygenation strategy with the pre-hospital equipment, or indeed the SOP they were working with. However, where discordance did exist it seemed to regard the utilisation of PEEP, either through BVM or Mapleson C. With many services providing the option of PEEP and around a third suggesting availability of a Mapleson C circuit, it raises stimulating discussion regarding variation in equipment availability, governance and oxygenation strategies amongst different services and whether these should exist. The carriage of Mapleson C circuits could be related to clinician familiarity and tactile feedback in terms of delivering PEEP and manual ventilation. It should be noted that all services carrying Mapleson C circuits also carried BVMs.

Although apnoeic oxygenation strategies were mentioned in all but one service SOP, recommendations for apnoeic oxygenation strategies appeared far less firm than for pre-oxygenation. In contrast to pre-oxygenation, a majority of services did not mandate the use of apnoeic oxygenation during PHEA. This may reflect the current evidence base for apnoeic oxygenation and its practical difficulties in the pre-hospital environment [24, 31]. It is interesting to note that many strategies advised nasal cannula with flow rates > 4 L/min and many clinicians reported their preference desire to deliver humidified high flow nasal oxygen, whilst no clinician or SOP reported use of non-invasive ventilation. All these strategies have resource implications, most significantly high flow nasal oxygen and non-invasive ventilation, requiring large amounts of oxygen and equipment that may limit their application to the pre-hospital environment. This heterogeneity in clinical practice highlights an area of uncertainty that merits further research and discussion.

Notably, 95% of clinicians reported manually ventilating patients during the apnoeic phase of PHEA. This practice is at odds with the description of traditional RSI [32] and the practice of many in-hospital clinicians [33]. Manual ventilation during RSI has remained a contentious topic, with some believing avoidance of mask ventilation is dogmatic practice [34]. There is mounting evidence in favour of mask ventilation in RSI [35], and this is reflected in practice guidelines [16]. The current UK pre-hospital practice revealed by this survey may be due to rapid translation of research into practice and clinician desire to practice evidence-based medicine.

This desire to capture and understand the reality of UK practice was reflected in our study design and mirrored in the pragmatic approach that we took to pre-oxygenation and apnoeic oxygenation definitions. Present literature definitions of apnoeic oxygenation are variable and imprecise [14, 16, 24, 36]. Often, apnoeic oxygenation is defined as oxygenation post administration of muscle relaxant during RSI [16, 36]. However, real-world application involves a secondary supply of oxygen, through an alternative route, usually by nasal cannula that remains in place during airway instrumentation [24, 31]. For this reason, our questions were targeted to oxygenation strategies pre and per airway instrumentation. Our findings suggest that many practitioners and services had a preference for two modalities of oxygen delivery pre-airway instrumentation, with one modality remaining in place during airway instrumentation to deliver apnoeic oxygenation. Better understanding of practical delivery of PHEA oxygenation and clarification of this nuance may assist in future description, comparison of practice, and standardisation of future nomenclature.

Our study achieved an excellent response rate covering all UK HEMS services. The results therefore likely represent a good summary of contemporaneous practice. The survey methodology presents risks of social acceptability and recall bias, however obtaining service SOP copies where possible helped to mitigate this. Nevertheless, participant responses may not represent real-life clinical practice. Whilst this survey has provided a comprehensive assessment of current UK practice, it does not assess clinical outcomes and hence further clinical studies investigating patient outcomes are needed. The heterogeneity in clinical practice highlighted by this study emphasises that the optimal pre-oxygenation strategy remains unclear and there appears to be uncertainty in the value of apnoeic oxygenation, and hence more detailed clinical studies are warranted.

Without understanding existing clinical practice we are unable to identify where practice is routine or divergent, and therefore where to prioritise research and focus to improve patient care. Whilst it is clearly important that individual services need to tailor care to meet their patients’ needs and, within those services, clinicians may need the flexibility to tailor strategies to meet the needs of each individual patient, we believe our study contributes to the national dialogue, highlights the benefits of sharing data and practice and thus, ultimately, allows shared learning across the speciality.

## Conclusions

This survey of all UK HEMS services identifies that pre-oxygenation is almost universal in current PHEA practice and most frequently delivered by bag valve mask without PEEP or non-rebreathable face masks. Conversely, use of apnoeic oxygenation is not widespread and there is significant variability in both individual clinician practice and service SOPs. Many clinicians are routinely ventilating patients during the apnoeic phase, whilst others are ventilating only in high risk patients. Differences between in-hospital and pre-hospital practice were principally due to availability of equipment such as humidified high flow nasal oxygenation and Mapleson C circuits. Multiple services carry bag valve masks with PEEP and/or Mapleson C circuits, however many services carry neither therefore precluding delivery of PEEP. All those carrying Mapleson C circuits also carried bag valve masks with or without a PEEP valve. The identified variability in oxygenation strategies during PHEA warrants further study, including assessment of clinical endpoints.

## Supplementary information

**Additional file 1.**

## Data Availability

The datasets used and/or analysed during the current study are available from the corresponding author on reasonable request.

## Abbreviations

BVM: Bag Valve Mask
HEMS: Helicopter Emergency Medical Service
PEEP: Positive End Expiratory Pressure
PHEA: Prehospital Emergency Anaesthesia
RSI: Rapid Sequence Induction
SOP: Standard Operating Procedure
THRIVE: Transnasal Humidified Rapid-Insufflation Ventilatory Exchange
