# Supplementary material for: Oxygenation strategies prior to and during prehospital emergency anaesthesia in UK HEMS practice (PREOXY survey)
Source: Scand J Trauma Resusc Emerg Med. 2020 Oct 12;28:99. doi: 10.1186/s13049-020-00794-x (PMC7552361; doi:10.1186/s13049-020-00794-x)
Supplement: Supplementary file 1 — Additional file 1. [file 13049_2020_794_MOESM1_ESM.docx]

**HEMS service SOP**

This relates to the HEMS service you are collecting for and their stated SOP. It would be very helpful if you are able to forward a copy of the service SOP to the study team (preoxysurvey@gmail.com).

PHEA = Pre-Hospital Emergency Anaesthesia

RSI = Rapid Sequence Induction

For the purposes of this study the delineation of oxygenation PRIOR to and DURING airway instrumentation are used and we would ask you to answer the questions with this in mind

| Which service are you responding on behalf of? | Drop-down list of all UK HEMS |
| --- | --- |
| What predominant model does your service use when delivering PHEA? | Doctor-paramedic team  Paramedic-paramedic team  Both |
| Is PHEA delivered by paramedic-paramedic teams within your service? | YES  NO |
| Approximately how many episodes are PHEA are delivered by your service per year? | <50  50-100  >100 |
| Does the HEMS service have a *Rapid Sequence Intubation (RSI) or Pre-Hospital Emergency Anaesthesia (PHEA) SOP*? | YES  NO |
| How does the HEMS service refer to pre-oxygenation?  (Pre-oxygenation: Strategies to oxygenate the patient prior to and during delivery of pre-hospital emergency anaesthesia, i.e. PRIOR TO airway instrumentation). | Standalone SOP  Part of RSI or PHEA SOP  Not referred to in any SOP |
| What pre-oxygenation strategies are explicitly stated in the SOPs? (Please state specific facemasks in other)  (Pre-oxygenation: Strategies to oxygenate the patient prior to and during delivery of pre-hospital emergency anaesthesia, i.e. PRIOR TO airway instrumentation). | Non-rebreathable face mask  Bag-valve-mask (BVM) *without* PEEP  Bag-valve-mask (BVM) *with* PEEP  Mapleson C  Non-invasive ventilation (NIV)  Nasal cannula, low flow (max 4L)  Nasal cannula, high flow (>4L)  No strategy stated in any SOP  Not referred to in any SOP  Other… |
| How is pre-oxygenation referred to in the SOP?  (Pre-oxygenation: Strategies to oxygenate the patient prior to and during delivery of pre-hospital emergency anaesthesia, i.e. PRIOR TO airway instrumentation). | Mandatory  Advised  Clinician judgement  Not referred to in any SOP  Other… |
| As required, please provide further details to elaborate upon your answer above | Long-answer text |
| Please state any specific patient groups in which the SOP states pre-oxygenation should be used (e.g. pregnant, obese). | Long-answer text |
| How does the HEMS service refer to oxygenation DURING airway instrumentation in PHEA? | Part of RSI or PHEA SOP  Standalone SOP  Not referred to in any SOP |
| What oxygenation strategies DURING airway instrumentation (e.g. laryngoscopy) are explicitly stated in the SOPs? | Nasal cannula, low flow (max 4L)  Nasal cannula, high (>4L)  No strategy stated in any SOP  Other… |
| How are oxygenation strategies DURING airway instrumentation (e.g. laryngoscopy) referred to in the SOP? | Mandatory  Advised  Clinician judgement  Not referred to in any SOP  Other… |
| Please state any specific patient groups in which the SOP states oxygenation DURING airway instrumentation should be used (e.g. pregnant, obese, all) | Long-answer text |

**Individual practice**

The following questions relate to your individual practice. Please answer these questions as accurately as possible. Your responses are anonymous and will not be identifiable in subsequent analysis and publication.

During the survey the acronym PHEA is used to denote Pre-Hospital Emergency Anaesthesia and also RSI to denote Rapid Sequence Induction.

The survey relates to the oxygenation process before and during PHEA. For the purposes of this study the delineation of oxygenation PRIOR to and DURING airway instrumentation are used and we would ask you to answer the questions with this in mind.

| Which service are you responding on behalf of? (will not be used for analysis, solely to ensure response rate) | Drop-down list of all UK HEMS |
| --- | --- |
| Approximate years of experience as a doctor | Drop-down list |
| Approximate years experience in pre-hospital emergency medicine | Drop-down list |
| Grade | Trainee  Consultant (not current in PHEM training)  Other… |
| Do you use pre-oxygenation?  (Pre-oxygenation: Strategies to oxygenate the patient prior to and during delivery of pre-hospital emergency anaesthesia, i.e. PRIOR TO airway instrumentation). | Always (100%)  Very often (75-100%)  Often (50-75%)  Occasionally (25-50%)  Rarely (0-25%)  Never (0%) |
| Which pre-oxygenation strategy do you most frequently use? (Please state specific facemasks in 'other')  (Pre-oxygenation: Strategies to oxygenate the patient prior to and during delivery of pre-hospital emergency anaesthesia, i.e. PRIOR TO airway instrumentation). | Non-rebreather face mask  Bag-valve-mask (BVM) *without* PEEP  Bag-valve-mask (BVM) *with* PEEP  Mapleson C  Non-invasive ventilation (NIV)  Nasal cannula, low flow (max 4L)  Nasal cannula, high flow (>4L)  Don’t use pre-oxygenation strategies  Other… |
| Does your oxygenation strategy change between delivering the anaesthetic drugs but before airway instrumentation. (If so please give as much detail as possible, e.g. will deliver some ventilation breaths to paralysed patient with BVM if criteria x met) | Long-answer text |
| Are you able to deliver your preferred pre-oxygenation strategy in your current service? (Pre-oxygenation: Strategies to oxygenate the patient prior to and during delivery of pre-hospital emergency anaesthesia, i.e. PRIOR TO airway instrumentation). | YES  NO |
| If no, what are the barriers to not being able to deliver your preferred oxygenation strategy PRIOR TO airway instrumentation (i.e. laryngoscopy)? Please select all that apply | Access to equipment  Alternative strategy mandated by SOP  Other… |
| Do you use oxygenation strategies DURING airway instrumentation (i.e. laryngoscopy) in PHEA? | Always (100%)  Very often (75-100%)  Often (50-75%)  Occasionally (25-50%)  Rarely (0-25%)  Never (0%) |
| For the last 5 PHEA you have delivered, what frequency have you used oxygenation strategies DURING airway instrumentation (i.e. laryngoscopy)? | 5 times  4 times  3 times  2 times  1 time  None |
| What oxygenation strategies DURING airway instrumentation (i.e. laryngoscopy) do you most frequently use? | Nasal cannula, low flow (max 4L)  Nasal cannula, high flow (>4L)  Don’t use oxygenation strategies during laryngoscopy  Other… |
| Are you able to deliver your preferred oxygenation strategy DURING airway instrumentation (i.e. laryngoscopy) in your current service? | YES  NO |
| If no, what are the barriers to not being able to deliver your preferred oxygenation strategy DURING airway instrumentation (i.e. laryngoscopy)? Please select all that apply | Access to equipment  Alternative strategy mandated by SOP  Other… |
| Please describe your ideal oxygenation strategy prior to and during PHEA | Long-answer text |
| Please describe your ideal in-hospital oxygenation strategy (please specify your in-hospital speciality) | Long-answer text |
| How do your PHEA and in-hospital practices differ? | Long-answer text |
